# Increased Expression of TRPS1 Affects Tumor Progression and Correlates with Patients' Prognosis of Colon Cancer

**DOI:** 10.1155/2013/454085

**Published:** 2013-05-26

**Authors:** Jun Hong, Jie Sun, Tao Huang

**Affiliations:** ^1^Department of General Surgery, Shanghai Putuo District Center Hospital, Shanghai 200062, China; ^2^Department of General Surgery, Shanghai Zhabei District Shibei Hospital, Shanghai 200435, China; ^3^Department of General Surgery, Shanghai Eighth People's Hospital, Shanghai 200235, China

## Abstract

*Aim*. To detect the expression pattern of tricho-rhino-phalangeal syndrome-1 (TRPS1) in human colon cancer and to analyze its correlation with prognosis of patients with this disease. *Methods*. The expressions of TRPS1 in human colon cancer and its corresponding noncancerous colon tissues were detected at both mRNA and protein levels. *Results*. The mRNA and protein expression levels of TRPS1 were both significantly higher in colon cancer than in corresponding noncancerous colon tissues (both *P* < 0.001). The protein level of TRPS1 in colon cancer tissues was significantly correlated with the mRNA level (*r* = 0.9, *P* < 0.001). Additionally, immunohistochemistry analysis also found increased TRPS1 expression in 63.0% (63/100) of colon cancer tissues. High TRPS1 expression was significantly associated with positive lymph node metastasis (*P* = 0.006) and higher pathological stage (*P* = 0.008) of patients with colon cancer. Multivariate Cox regression analysis further suggested that the increased expression of TRPS1 was an independent poor prognostic factor for this disease. *Conclusion*. Our data offer the convincing evidence for the first time that the increased expression of TRPS1 may be involved in the pathogenesis and progression of colon cancer. TRPS1 might be a potential marker to predict the prognosis in colon cancer.

## 1. Introduction

Colon cancer represents the third most common cancer worldwide and the most common malignancy of the gastrointestinal tract [1]. Although the diagnostic and therapeutic technologies have been improved greatly, the morbidity and mortality of colon cancer have increased in recent years. There are over one million cases with this disease occurring annually [2]. The mortality rate for colon cancer is approximately half of its global incidence [3]. Endoscopy remains currently the most important screening procedure with therapeutic value for colon cancer. However, prognostic markers for patients with this disease lack specificity and sensitivity. Some molecular mechanisms of colonic carcinogenesis have been explained, but factors affecting the progression remain unclear. Thus, it is of great significance to investigate the molecular events involved in colon cancer onset and aggressive progression in order to improve levels of early detection and treatment.

Tricho-rhino-phalangeal syndrome-1 (TRPS1), an atypical member of the GATA-type family of transcription factors, is involved in TRPS, an autosomal dominant skeletal disorder [4]. The TRPS1 gene is approximately 260.5 kb in length and consists of 7 exons. The third exon contains a Kozak consensus ATG translation start site and the first C_2_H_2_-type zinc finger domain. The seventh exon encodes an Ikaros-type zinc finger domain and a TAA stop codon [5]. TRPS1 encodes a 1281-amino-acid (aa) zinc-finger transcription factor that has a calculated molecular mass of 160 kDa and contains an unusual combination of nine predicted zinc finger domains, including seven classical C_2_H_2_-type domains, which are related to those found in the transcription factor TFIIIA of Xenopus laevis, one GATA C4-type domain, and two Ikaros C_2_H_2_-type zinc fingers [6]. The GATA zinc finger is flanked by two basic nuclear localization signals (NLS1 and NLS2). It has been demonstrated that only the second motif functions as an NLS in TRPS1 suggesting that TRPS1 may act as a nuclear zinc finger protein [7]. Under physiological conditions, TRPS1 expression has been found to be high in the prostate, testis, ovary, kidney, lung, and mammary gland. Lower levels of its expression have been found in the liver, colon, heart, uterus, and brain [8]. More interestingly, its expression in fetal tissues (kidney, lung, liver, heart, and brain) has been found to be lower than that in the corresponding adult tissues, suggesting that during adulthood TRPS1 might be involved in growth suppression of these tissues [9]. Recent studies have demonstrated that TRPS1 may exhibit a variety of functions in human malignancies. The TRPS1 gene is localized on human chromosome 8q23-24.1, a region highly amplified in several cancers, especially prostate cancer and breast cancer, indicating that TRPS1 may be overexpressed during development or progression of these endocrine-related cancers [10]. However, its roles in colon cancer are still unclear. The aim of this study was to detect the expression pattern of TRPS1 in human colon cancer and to analyze its correlation with prognosis of patients with this disease. 

## 2. Materials and Methods

### 2.1. Patients and Tissue Samples

This study was approved by the Research Ethics Committee of Shanghai Zhabei District Shibei Hospital, China. Written informed consent was obtained from all of the patients. All specimens were handled and made anonymous according to the ethical and legal standards.

For qRT-PCR and Western blot analysis, 40 fresh specimens including 20 pairs of colon cancer (aging 52 ~ 86 years, mean ± SD = 63.9 ± 12.3 years, TNM staging from I to IV) and their matched noncancerous colon tissues were frozen in liquid nitrogen immediately after surgical resection for extraction of RNA and protein. For immunohistochemistry, 100 tumor tissues from 100 patients with colon cancer who had undergone surgery at Shanghai Zhabei District Shibei Hospital, between 1994 and 2003, were collected. None of the patients received radiotherapy, chemotherapy, or other medical interventions prior to surgical operation. A pair of tissue samples for each case was collected from the tumor tissues and their corresponding noncancerous colon tissues. The clinicopathological characteristics were determined according to the classification of malignant tumors by the World Health Organization and International Union against Cancer Tumor-Node-Metastasis (TNM) staging system. The clinical and pathologic parameters were obtained from the pathological reports and presented in Table 1. All the patients were followed up from the excision of the primary tumor until death, or the closing date of this study (October 19, 2012). The median followup for all the patients was 53 months (range, 2–93 months).

### 2.2. QRT-PCR Analysis

QRT-PCR was performed to detect the expression level of TRPS1 mRNA in 20 pairs of primary colon cancer and corresponding noncancerous colon tissues using SYBR Green Real-Time PCR Master Mix (Toyobo, Japan). The forward and reverse primers were 5′-GTA TCC TGC ATC GGG AGA AA-3′ and 5′-AGC TTC TGG TAG AGG CCA CA-3′ for the target gene TRPS1 and were 5′-CTT AGT TGC GTT ACA CCC TTT CTT G-3′ and 5′-CTG TCA CCT TCA CCG TTC CAG TTT-3′ for the reference gene *β*-actin. qPCR was performed in triplicates, and each 20 *μ*L reaction contained 2× SYBR Green Master Mix (Toyobo, Japan), 1 *μ*L forward and reverse primers, and 2 *μ*L of cDNA template. The melting curve analysis was set to determine the specificity of candidate gene amplification. Data were analyzed through the comparative threshold (CT) cycle method.

### 2.3. Western Blot Analysis

Western blot analysis was performed to detect the expression level of TRPS1 mRNA in 20 pairs of primary colon cancer and corresponding noncancerous colon tissues. Briefly, protein extracts (30 *μ*g) were prepared with RIPA lysis buffer (50 mM Tris-HCl, 150 mM NaCl, 0.1% SDS, 1% NP-40, 0.5% sodium deoxycholate, 1 mM PMSF, 100 mM leupeptin, and 2 mg/mL aprotinin, pH 8.0). Protein concentration was determined by Bradford assay. The extracted proteins were resolved by a 10% SDS-PAGE gel and transferred onto an Immobilon-P PVDF transfer membrane (Millipore, USA) by electroblotting. After blocking with 5% nonfat milk, the membranes were probed with rabbit anti-TRPS1 polyclonal antibody (no. ab111439, Abcam, Cambridge, MA, USA). Then, the blots were incubated with peroxidase-conjugated anti-rabbit IgG (KPL, USA) for 1 h at room temperature at a 1 : 3000 dilution and then developed by using the ECL kit (SuperSignal West Pico Trial Kit, Thermo Scientific, USA).

### 2.4. Immunohistochemistry Analysis

The subcellular localization and expression patterns of TRPS1 were detected by immunohistochemistry analysis on formalin-fixed, paraffin-embedded, 4 *μ*m thick tissue sections using the avidin-biotin-peroxidase complex method. Briefly, following a brief proteolytic digestion and a peroxidase blocking of tissue slides, the slides were incubated with the primary antibody against TRPS1 (rabbit anti-TRPS1, 1 : 100; no. ab111439, Abcam, Cambridge, MA, USA) overnight at 4°C. After washing, peroxidase labeled polymer and substrate-chromogen were then employed in order to visualize the staining of the interested proteins. In each immunohistochemistry run, negative controls were carried out by omitting the primary antibody, whereas TRPS1 overexpression confirmed by Western blotting was used as positive controls.

Following a hematoxylin counterstaining, immunostaining was scored by two independent experienced pathologists, who were blinded to the clinicopathological parameters and clinical outcomes of the patients. Given the homogeneity of the staining of the target proteins, tumor specimens were scored in a semiquantitative manner. The scores of the two pathologists were compared and any discrepant scores were trained through reexamining the stainings by both pathologists to achieve a consensus score. The percentage scoring of immunoreactive tumor cells was as follows: 0 (0%), 1 (1%–10%), 2 (11%–50%), and 3 (>50%). The staining intensity was visually scored and stratified as follows: 0 (negative), 1 (weak), 2 (moderate), and 3 (strong). A final immunoreactivity score (IRS) was obtained for each case by multiplying the percentage and the intensity score. Protein expression levels were further analyzed by classifying IRS values as low (based on an IRS value lower than 4.5 which was the median of IRS values) and as high (based on an IRS value greater than 4.5 which was the median of IRS values). 

### 2.5. Statistical Analysis

The software of SPSS version 13.0 for Windows (SPSS Inc, IL, USA) as well as SAS 9.1 (SAS Institute, Cary, NC) was used. Differences of TRPS1 expression at mRNA and protein levels between colon cancer and paired noncancerous colon tissues were analyzed by paired samples *t*-test. In addition, statistical analyses were performed with Fisher's exact test for any 2 × 2 tables, Pearson *χ* ^2^ test for non-2 × 2 tables, and Kaplan-Meier and Cox Regression methods for the question of survival analysis. Spearman rank correlation is used to analyze the correlation between the expression of TRPS1 mRNA and protein. Differences were considered statistically significant when *P* value was less than 0.05. The *P* values in this study had not been adjusted for multiple comparisons.

## 3. Results

### 3.1. Expression of TRPS1 mRNA and Protein in Human Colon Cancer Tissues

**Figure 1:**
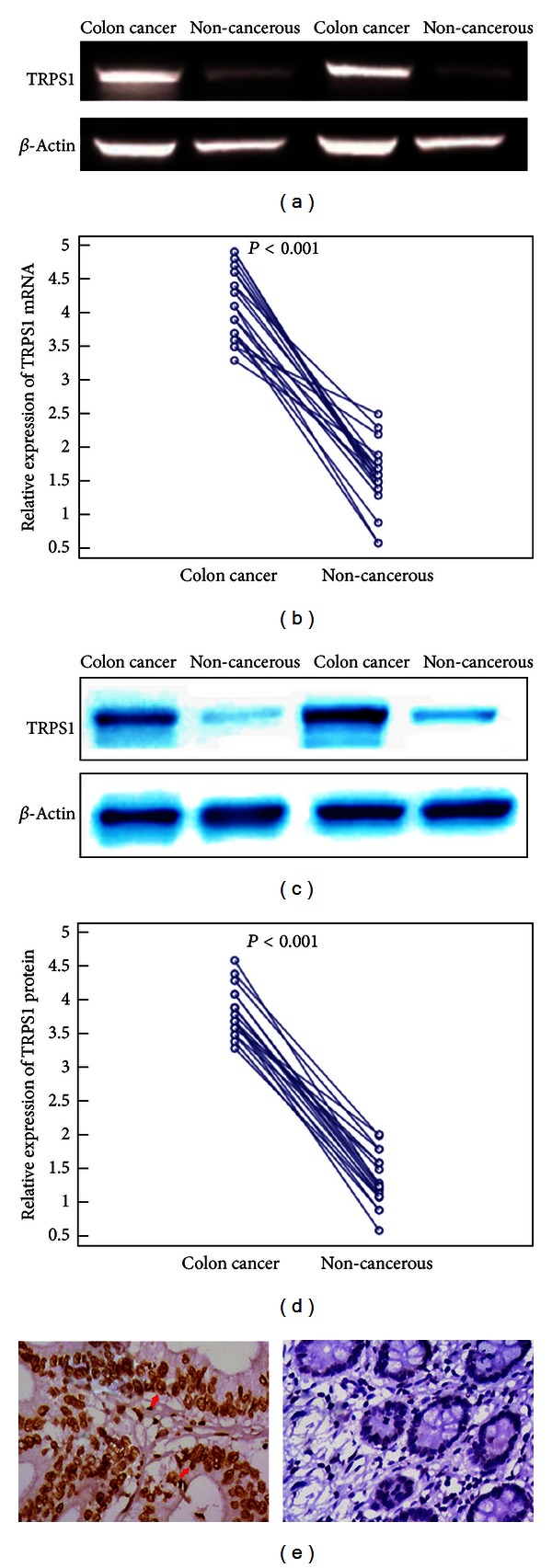
Expression of TRPS1 mRNA and protein in human colon cancer tissues. (a) Representative photograph of qRT-PCR analysis. (b) Relative expression ratio of TRPS1 mRNA to *β*-actin mRNA. (c) Representative blots of Western blot analysis. (d) Relative expression ratio of TRPS1 protein to *β*-actin protein. (e) Immunohistochemical expression for TRPS1 in colon cancer and paired noncancerous colon tissues (×200). TRPS1 was strongly expressed in the nucleus of colon cancer cells (left), but not detected in the noncancerous colon tissues (right).

The expression of TRPS1 mRNA in 20 pairs of colon cancer and their matched noncancerous colon tissues was detected by qRT-PCR. We found that TRPS1 mRNA was expressed at a high level in colon cancer tissues compared with matched noncancerous colon tissues (4.1 ± 0.5 versus 1.6 ± 0.5, *P* < 0.001, Figures 1(a) and 1(b)). To investigate whether TRPS1 upregulation was also apparent at the protein level, we performed Western blot analysis of TRPS1 protein in these 20 paired samples. We found that the observations in Western blot analysis were in agreement with those in qRT-PCR. Representative blots were shown in Figure 1(c). Statistical analysis also indicated that the protein expression levels of TRPS1 were significantly higher in colon cancer tissues compared with their normal counterparts (3.8 ± 0.3 versus 1.3 ± 0.4, *P* < 0.001, Figure 1(d)). The Spearman rank correlation analysis revealed a significant correlation between the expression of TRPS1 mRNA and protein (*r* = 0.9, *P* < 0.001).

To locate TRPS1 protein expression in human colon cancer tissues, immunohistochemistry was performed. TRPS1 was expressed in the nucleus of colon cancer cells in 63/100 (63.0%, Figure 1(e)) of cases with unequal intensity. By contrast, the immunoreactivity of this protein was not detected in the noncancerous colon tissues.

### 3.2. Correlation of TRPS1 Expression with the Clinicopathological Characteristics of Human Colon Cancer

In order to analyze the correlation of TRPS1 expression with various clinicopathological characteristics of patients with colon cancer, we divided 100 patients into two groups according to the expression levels of TRPS1 protein detected by immunohistochemistry. Colon cancer patients with IRS of TRPS1 protein more than the median expression level (4.5) were assigned to the high TRPS1 expression group (mean expression value 5.2, *n* = 56), and those samples with IRS equal or below the median value were assigned to the low TRPS1 expression group (mean expression value 3.8, *n* = 44). As shown in Table 1, the high TRPS1 expression was significantly associated with positive lymph node metastasis (*P* = 0.006) and higher pathological stage (*P* = 0.008) of patients with colon cancer. However, TRPS1 exhibited no significant association with other clinicopathological characteristics of colon cancer, such as the age, gender, tumor size, or differentiation (all *P* > 0.05).

### 3.3. Correlation of TRPS1 Expression with the Prognosis in Patients with Colon Cancer

**Figure 2:**
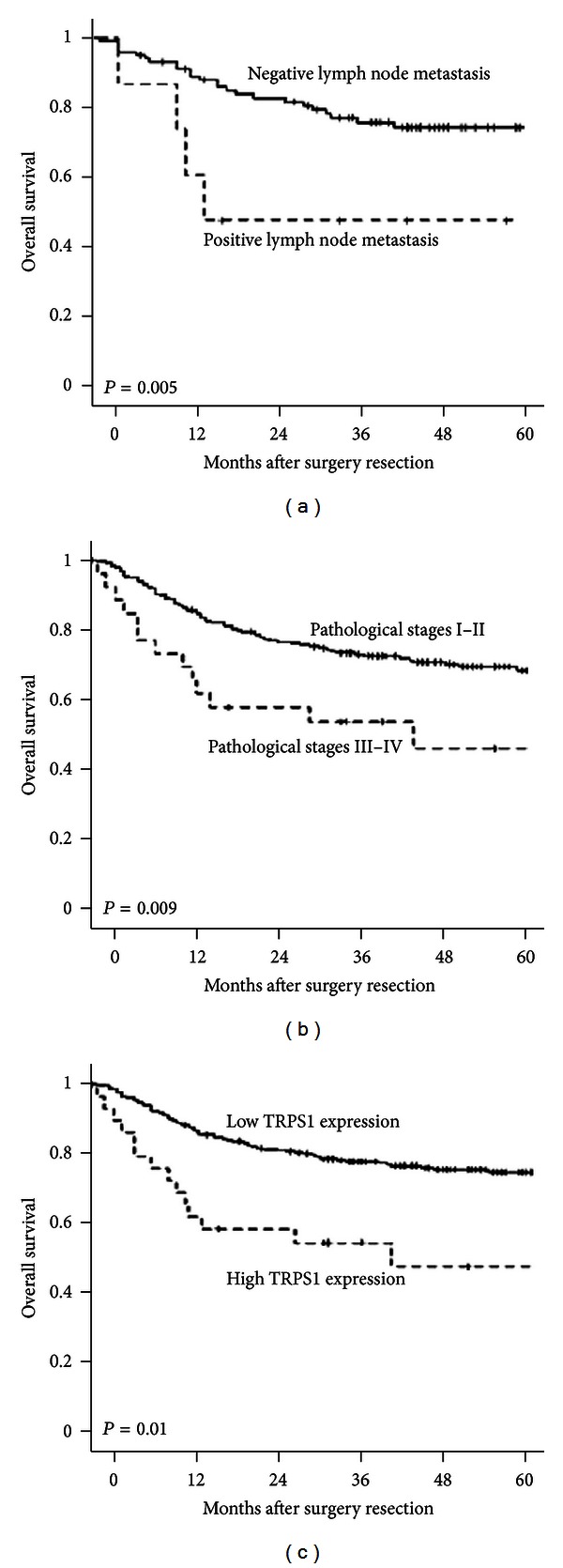
Kaplan-Meier survival curves of patients with colon cancer according to lymph node metastasis (a), pathological stage (b), and the expression of TRPS1 (c).

Kaplan-Meier survival analysis revealed that lymph node metastasis (*P* = 0.005, Figure 2(a)) and pathological stage (*P* = 0.009, Figure 2(b)) were significantly associated with the overall survival in the patients. The overall survival in patients with high TRPS1 expression was significantly shorter than in those with low TRPS1 expression (*P* = 0.01, Figure 2(c)). Cox multivariate analysis showed that lymph node metastasis (*P* = 0.01) as well as pathological stage (*P* = 0.02), and TRPS1 expression (*P* = 0.03) in colon cancer were negatively correlated with postoperative overall survival, and positively correlated with mortality, suggesting that TRPS1 may be a relative risk factor for prognosis in patients with colon cancer (Table 2).

## 4. Discussion

The occurrence and progression of human cancers have been demonstrated to be associated with a series of genetic events affecting the structure and/or the expression of a number of oncogenes and antioncogenes. However, the molecular mechanisms of carcinogenesis in colon cancer have not been fully elucidated. In the current study, we detected the expression of TRPS1 at both mRNA and protein levels and then explored its prognostic value by using complete long-term follow-up data of a cohort of colon cancer samples. The results showed that TRPS1 mRNA and protein expression were both significantly upregulated in colon cancer tissues as compared with their corresponding noncancerous colon tissues. In addition, tissue immunohistochemistry analysis indicated that TRPS1 expression was increased in more than half of the primary colon cancer tissues but was not detected in all noncancerous colon tissues (all IRS values were 0). TRPS1 upregulation was significantly associated with higher pathological stage and positive lymph node metastasis. Furthermore, colon cancer patients with high TRPS1 expression had shorter overall survival than those with low TRPS1 expression. Collectively, our data suggest that TRPS1 might represent a novel indicator for the prognosis of colon cancer. 

TRPS1 is a complex and multitype zinc-finger protein according to the results of molecular cloning analysis [11]. Many proteins containing zinc-finger structures have been demonstrated to be involved in oncogenesis. Rahman et al. [12] found that GATA-4 mRNA expression was markedly upregulated in murine adrenal tumors as well as in human adrenocortical carcinomas, whereas it was undetectable in normal adrenal cortex during mouse development. As TRPS1 has a GATA domain, many researchers have hypothesized that it might play some roles in tumorigenesis in human tissues. Chang et al. [13] in 2000 indicated that TRPS1 was potentially involved in apoptosis, which may have implications for the development of androgen-independent prostate cancer and ultimately for the treatment of this disease. In 2004, the same group found that TRPS1 protein expression was downregulated by androgens in human prostate cancer, and analysis of TRPS1 mRNA expression levels in several human tissues showed that the highest levels were observed in normal and cancerous breast tissues [14]. After that, they also demonstrated that the overexpression of TRPS1 protein was correlated with reduced protein expression of certain antioxidants, suggesting a possible involvement of TRPS1 in oxidative stress and possibly in apoptosis in androgen-independent DU145 prostate cancer cells [15]. Stinson et al. [16] further indicated that TRPS1 inhibited epithelial-to-mesenchymal transition in breast cancer by directly repressing expression of zinc-finger E-box-binding homeobox 2. On the basis of these findings, our data here also revealed the clinical relevance of TRPS1 expression to progression and prognosis of colon cancer. However, the mechanisms by which TRPS1 is aberrantly expressed in colon cancer need further investigation.

In conclusion, our data offer the convinced evidence for the first time that the increased expression of TRPS1 may be involved in the pathogenesis and progression of colon cancer. TRPS1 might be a potential marker to predict the prognosis in colon cancer. This is the first report to suggest a relationship between TRPS1 overexpression and prognosis in patients with colon cancer, and further prospective analysis would be worth doing.

## Figures and Tables

**Table 1:** Correlations of TRPS1 expression with the clinicopathological features of colon cancer.

Clinicopathological features	No. of cases (%)	TRPS1		
		High (%)	Low (%)	*P*
Age (years)				
<60	41 (41.0)	23 (56.1)	18 (43.9)	NS
≥60	59 (59.0)	33 (55.9)	26 (44.1)	
Gender				
Female	52 (52.0)	32 (61.5)	20 (38.5)	NS
Male	48 (48.0)	24 (50.0)	24 (50.0)	
Tumor size (cm)				
≤5	65 (65.0)	39 (60.0)	26 (40.0)	NS
>5	35 (35.0)	17 (48.6)	18 (51.4)	
Lymph node metastasis				
Negative	46 (46.0)	12 (26.1)	34 (73.9)	0.006
Positive	54 (54.0)	44 (81.5)	10 (18.5)	
Pathological stage				
I	33 (33.0)	2 (25.5)	31 (74.5)	0.008
II	51 (51.0)	38 (74.5)	13 (25.5)	
III	16 (16.0)	16 (100)	0 (0)	
Differentiation				
Well	16 (16.0)	9 (56.3)	7 (43.7)	NS
Moderately	52 (52.0)	27 (51.9)	25 (48.1)	
Poorly	32 (32.0)	20 (62.5)	12 (37.5)	

Note: “NS” refers to the differences without statistical significance.

**Table 2:** Multivariate analysis of prognostic factors in patients with colon cancer.

Prognostic factors	Relative risk (95% confidence interval)	*P*
Tumor size	1.51 (0.72–3.73)	0.32
Lymph node metastasis	5.68 (1.82–13.62)	0.01
Pathological stage	4.79 (1.53–11.69)	0.02
Differentiation	1.52 (0.73–3.67)	0.46
TRPS1 expression	4.09 (1.46–11.06)	0.03
